# Verhaltensdaten zu klimagesundem Verhalten nutzbar machen: Methoden, Potenziale und Perspektiven

**DOI:** 10.1007/s00103-025-04106-5

**Published:** 2025-08-05

**Authors:** Dominik Daube, Mirjam A. Jenny, Sarah Pelull

**Affiliations:** 1 Institute for Planetary Health Behaviour, Gesundheitskommunikation, Universität Erfurt, Nordhäuser Str. 63, 99089 Erfurt, Deutschland; 2 Arbeitsgruppe Gesundheitskommunikation, Implementation, Bernhard-Nocht-Institut für Tropenmedizin, Hamburg, Deutschland; 3 Forschungsbereich für adaptive Rationalität, Max-Planck-Institut für Bildungsforschung, Berlin, Deutschland; 4 Harding-Zentrum für Risikokompetenz, Universität Potsdam, Potsdam, Deutschland

**Keywords:** Klimagesunde Routinen, COM-B-Framework, Behavioural and Cultural Insights, Mixed-Methods-Ansatz, Gesundheitsförderung, Climate-healthy routines, COM-B framework, Behavioural and cultural insights, Mixed-methods approach, Health promotion

## Abstract

Klimagesundes Verhalten nimmt eine Schlüsselposition in der Gestaltung nachhaltiger Strategien zur Gesundheitsförderung ein. Dieser Beitrag zeigt, wie Verhaltensdaten genutzt werden können, um individuelle Routinen und strukturelle Rahmenbedingungen besser zu verstehen und wirksame Maßnahmen evidenzbasiert zu konzipieren. Auf Basis des interdisziplinären Ansatzes Behavioural and Cultural Insights (BCI) sowie des theoretischen COM-B-Frameworks (Capability, Opportunity, Motivation – Behaviour) wird erläutert, wie psychologische, soziale und kulturelle Faktoren das menschliche Verhalten beeinflussen und welche Rolle die systematische Verhaltensdatenerhebung für die Förderung klimagesunder Routinen spielt.

Einerseits erlauben quantitative Ansätze wie Online-Umfragen, Experience Sampling (Echtzeiterhebung von Erleben und Verhalten im Alltag) und (Online‑)Experimente die Analyse großer Stichproben und das Aufdecken von Verhaltensmustern. Andererseits liefern qualitative Methoden (z. B. Interviews, Tagebuchstudien) vertiefte Einblicke in Entscheidungsprozesse und soziale Normen. Zu sogenannten Mixed-Methods-Ansätzen zusammengeführt können diese Verfahren Barrieren identifizieren – etwa mangelndes Wissen oder unzureichende Infrastruktur – und gezielt adressieren.

Anhand der Projekte HEATCOM und PACE wird exemplarisch illustriert, wie Hitzeschutz- und klimagesundes Verhalten diesem integrativen Konzept folgend untersucht wird. Zugleich werden ethische Fragen sowie Aspekte des Datenschutzes diskutiert und aufgezeigt, wie Manipulationsvorwürfen durch Transparenz und wissenschaftliche Evidenz begegnet werden kann. Abschließend werden Herausforderungen in der politischen Umsetzung erörtert und Handlungsempfehlungen für Entscheidungsträger*innen formuliert, um Verhaltensdaten erfolgreich in Maßnahmen zur Klimaanpassung und Gesundheitsförderung zu integrieren.

## Abstract (en)

Climate-healthy behaviour plays a key role in designing sustainable strategies for health promotion. This article demonstrates how behavioural data can be leveraged to gain a clearer understanding of individual routines and structural conditions, and how evidence-based measures can be developed on that basis. Based on the interdisciplinary approach of behavioural and cultural insights (BCIs) and the COM‑B theoretical framework (Capability, Opportunity, Motivation—Behaviour), it explains how psychological, social and cultural factors influence human behaviour and the role of systematic behavioural data collection in promoting climate-healthy routines.

On one hand, quantitative approaches such as online surveys, experience sampling (a real-time survey of experience and behaviour in everyday life) and (online) experiments allow for large-scale analysis and the identification of behavioural patterns. On the other hand, qualitative methods (e.g. interviews, focus groups, diary studies) provide in-depth insights into decision-making processes and social norms. Combined into so-called mixed-methods approaches, these procedures can uncover barriers—such as limited knowledge or insufficient infrastructure—and target them.

Using the HEATCOM and PACE projects as examples, the article illustrates how heat protection and climate-healthy behaviour are examined within this integrative concept. Ethical considerations and data protection issues are also discussed, demonstrating how transparency and scientific evidence can dispel concerns of manipulation. Finally, the challenges of political implementation are explored, and recommendations for decision-makers are outlined on how to successfully integrate behavioural data into climate adaptation and health promotion measures.

## Einleitung

Klimagesundes Verhalten ist ein zentraler Bestandteil der Planetary-Health-Perspektive und spielt eine entscheidende Rolle für die Gestaltung nachhaltiger Public-Health-Strategien [1–3]. Unter dem Begriff „klimagesundes Verhalten“ werden in der vorliegenden Arbeit 2 unterschiedliche, aber eng miteinander verknüpfte Bereiche zusammengefasst:

1. Klimaschutzverhalten, das darauf abzielt, den Ausstoß von Treibhausgasen zu reduzieren (z. B. klimafreundliche Mobilität, ressourcenschonende Ernährung), und
2. Klimaanpassungsverhalten, das den Schutz der eigenen Gesundheit vor den Auswirkungen des Klimawandels in den Mittelpunkt stellt (z. B. Hitzeschutzmaßnahmen).

Diese Bereiche verfolgen unterschiedliche Ziele und Maßnahmen, werden jedoch in Praxis und Diskussion oft vermischt, was die konzeptionelle Präzision erschwert. So wird hier bewusst eine integrative Perspektive eingenommen, die beide Teilbereiche berücksichtigt.

Konkrete klimagesunde Verhaltensmaßnahmen umfassen den Schutz vor Hitze, die Vorsorge gegenüber neuen Krankheitsrisiken (z. B. Schutz vor Vektoren wie Zecken/Mücken), eine ressourcenschonende und ausgewogene Ernährung (z. B. überwiegend pflanzenbasiert, regional) sowie klimafreundliche Mobilität (z. B. Nutzung öffentlicher Verkehrsmittel/Fahrrad). Außerdem zählen bewusstes Konsumverhalten, Energieeinsparungen im Haushalt und Engagement für Umwelt- und Gesundheitsbelange dazu.

Allerdings lässt sich das Verhalten von Individuen nicht isoliert betrachten, da dieses tief in sozialen, kulturellen und strukturellen Kontexten verwurzelt ist [4]. Bei der Förderung oder Hemmung klimagesunden Verhaltens spielen sowohl individuelle Faktoren, wie Wissen, Einstellungen und Gewohnheiten, als auch strukturelle Rahmenbedingungen, wie Infrastruktur und politische Maßnahmen, eine wesentliche Rolle [5–7].

Die Relevanz des Themas „klimagesundes Verhalten“ hat in den letzten Jahren weiter zugenommen, da extreme Wetterereignisse, die gesundheitlichen Auswirkungen des Klimawandels und die Notwendigkeit nachhaltiger Verhaltensänderungen zunehmend im Fokus von Forschung, Politik und Öffentlichkeit stehen.

Um klimagesundes Verhalten zu fördern, ist es essenziell, zugrunde liegende Verhaltensmuster und Barrieren zu verstehen. Dies ermöglicht der Ansatz der *Behavioural and Cultural Insights (BCI)*, der u. a. von der Weltgesundheitsorganisation (WHO) vorangetrieben wird [2, 4]. Der Ansatz betrachtet das Zusammenspiel psychologischer, sozialer, kultureller und umweltbezogener Faktoren, die das Verhalten beeinflussen [8]. Diese inter- und transdisziplinäre Perspektive ermöglicht eine integrative Betrachtung der Determinanten von Gesundheitsverhalten und eröffnet neue Möglichkeiten zur Entwicklung evidenzbasierter Interventionen im Bereich Public Health. Durch eine Analyse von bewussten und unbewussten Entscheidungsprozessen im Alltag können BCI dazu beitragen, nachhaltige Veränderungen anzustoßen, die sowohl auf individueller als auch auf gesellschaftlicher Ebene wirksam sind [4].

Ein entscheidender Bestandteil dieses Ansatzes ist die systematische Nutzung von *Verhaltensdaten*, um klimagesundes Verhalten besser zu verstehen [8]. Anders als beispielsweise rein epidemiologische Kennzahlen, die Ausmaß und Verbreitung von Krankheiten oder Risikofaktoren in der Bevölkerung abbilden, liefern Verhaltensdaten Einblicke in konkrete Handlungsweisen und Entscheidungsprozesse, die hinter dem Gesundheits- und Klimaverhalten stehen. Sie liefern wertvolle Erkenntnisse über die tatsächliche Umsetzung von gesundheits- und klimabezogenen Maßnahmen und ermöglichen eine evidenzbasierte Gestaltung von Public-Health-Interventionen [9, 10].

Unter Verhaltensdaten werden in diesem Kontext alle empirischen Daten verstanden, die direktes oder indirektes Verhalten von Individuen abbilden. Dazu zählen beispielsweise Selbstberichte zu tatsächlichem Verhalten (z. B. Angaben zur Nutzung klimafreundlicher Verkehrsmittel oder zu Hitzeschutzmaßnahmen), Verhaltensbeobachtungen, digitale Tracking-Daten, die Verhaltensmuster erfassen können (z. B. Standort- oder Bewegungsdaten in Mobilitätsstudien), oder experimentelle Studien, über die sich verschiedene Facetten und Determinanten des Verhaltens erfassen lassen. In der Praxis wird u. a. über standardisierte Fragen (z. B. „Wie oft haben Sie im letzten Monat das Fahrrad statt des Autos genutzt?“) oder Experience Sampling (Echtzeiterhebung von Erleben und Verhalten im Alltag) erhoben. Damit unterscheiden sich Verhaltensdaten klar von bloßen Einstellungs- oder Intentionserhebungen, da sie den Fokus auf tatsächliche Handlungen legen.

Darüber hinaus sind nicht nur die Verhaltenswissenschaften, sondern auch verhältnisorientierte Ansätze von zentraler Bedeutung. Während sich Erstere vor allem mit den psychologischen und sozialen Mechanismen individueller Entscheidungen befassen, betrachten Letztere übergeordnete strukturelle Rahmenbedingungen wie Infrastruktur, Politik und Umwelteinflüsse [11, 12]. Diese Unterscheidung ist insofern wesentlich, als klimagesundes Verhalten sowohl im persönlichen Alltag (Verhaltensebene) als auch durch Veränderungen im Umfeld (Verhältnisebene) gefördert werden kann.

In diesem Beitrag wird diskutiert, wie Verhaltensdaten systematisch erhoben und genutzt werden können, um klimagesundes Verhalten besser zu verstehen und zu unterstützen. Dabei werden methodische Ansätze vorgestellt, die Möglichkeiten und Grenzen der Nutzung von Verhaltensdaten diskutiert und konkrete Anwendungsbeispiele skizziert. Zudem wird die Bedeutung von BCI für die Entwicklung und Implementierung effektiver Maßnahmen zur Förderung klimagesunden Verhaltens hervorgehoben. Abschließend werden Empfehlungen für politische Entscheidungsträger*innen und zukünftige Forschungsansätze formuliert, um das Potenzial von Verhaltensdaten für Public-Health-Strategien optimal zu nutzen.

## Theoretische Perspektiven und methodische Ansätze

Die Untersuchung klimagesunden Verhaltens erfordert eine fundierte theoretische Grundlage, um Determinanten und Mechanismen des Verhaltens besser zu verstehen und geeignete Interventionsstrategien abzuleiten. Theoretische Modelle bieten dabei eine systematische Struktur, um individuelle, soziale und strukturelle Einflussfaktoren zu identifizieren und gezielt in Public-Health-Maßnahmen zu integrieren.

In diesem Kapitel werden zunächst exemplarisch 2 zentrale theoretische Ansätze vorgestellt – das *COM-B-Framework* und der *Public Health Action Cycle* –, die erklären, welche Bedingungen für die Förderung klimagesunden Verhaltens notwendig sind. Anschließend werden methodische Verfahren zur Erhebung von Verhaltensdaten diskutiert, die eine empirische Analyse dieser theoretischen Modelle ermöglichen. Die Kombination aus theoretischer Fundierung und empirischer Methodik erlaubt eine evidenzbasierte Gestaltung von Strategien und Interventionen.

### Theoretische Perspektiven: COM-B-Framework und Public Health Action Cycle

Die 2 zentralen Ansätze COM-B-Framework und der Public Health Action Cycle stellen eine systematische Verbindung zwischen individuellen klimagesunden Verhaltensweisen und strukturellen Faktoren her.

Das *COM-B-Framework* [13] beschreibt Verhalten über 3 Kernkomponenten (Tab. 1):

- *Capability (individuelle Fähigkeit): *individuelle kognitive und physische Voraussetzungen zur Umsetzung klimagesunden Verhaltens (z. B. Wissen über Hitzeschutzmaßnahmen oder körperliche Mobilität),
- *Opportunity (Gelegenheit und Kontext): *die äußeren Umstände, die das Verhalten ermöglichen oder erschweren (z. B. gesetzliche Regulierungen, Infrastrukturförderungen sowie soziale und kulturelle Normen),
- *Motivation (individuelle Motivation):* individuelle Anreize und Überzeugungen, die das Verhalten beeinflussen (z. B. wahrgenommene persönliche Betroffenheit durch Klimaveränderungen).

**Tab. 1:** Zusammenfassung des COM-B-Frameworks (theoretischer Ansatz zur Verhaltensänderung) und exemplarische Anwendung zur Förderung klimagesunden Verhaltens

Komponente	Beschreibung	Beispiele für Determinanten klimagesunden Verhaltens	Mögliche Interventionen
*Capability*	Physische und kognitive Fähigkeiten	Körperliche Mobilität, Wissen über Hitzeschutz	Informationskampagnen, Schulungen
*Opportunity*	Soziale und strukturelle Rahmenbedingungen	Vorhandensein klimafreundlicher Mobilitätsangebote	Gesetzliche Regulierung, Infrastrukturförderung
*Motivation*	Intrinsische und extrinsische Anreize	Persönliche Wahrnehmung von Klimarisiken, sozialer Druck	Nudging, Stärkung sozialer Normen

Diese 3 Komponenten (COM) interagieren miteinander und liefern praxisorientierte Hinweise darauf, ob bzw. unter welchen Bedingungen eine Person ein bestimmtes Verhalten (Behaviour, B) zeigt. So lassen sich aus dem Modell COM‑B verschiedene Prädiktoren und Determinanten für gesundheitsrelevante und klimagesunde Verhaltensweisen ableiten, was eine gezielte Identifikation von Barrieren und Potenzialen für Interventionen ermöglicht.

Der *Public Health Action Cycle* [14–16] bietet eine ergänzende Perspektive, indem er den Prozess der Entwicklung und Implementierung von Public-Health-Maßnahmen in 4 Phasen unterteilt (Tab. 2):

1. *Assessment (Problembestimmung):* Identifikation von Problemen und relevanten Einflussfaktoren durch Verhaltensanalysen,
2. *Policy Formulation/Development (Strategieformulierung):* Gestaltung zielgerichteter Interventionen, basierend auf den gewonnenen Erkenntnissen,
3. *Assurance/Implementation (Umsetzung):* Anwendung der Maßnahmen in der Praxis unter Berücksichtigung sozialer und politischer Kontexte,
4. *Evaluation (Bewertung):* Überprüfung der Effektivität und Anpassung der Maßnahmen, um langfristige Verhaltensänderungen zu fördern.

**Tab. 2:** Phasen des Public Health Action Cycle (theoretischer Ansatz zum Ablauf von Gesundheitsinterventionen) mit Bezug auf klimagesundes Verhalten. Die relevanten Methoden sind nicht erschöpfend dargestellt und geben einen Überblick. Sie sind auch über mehrere Phasen hinweg anwendbar

Phase	Ziel	Bezug zu klimagesundem Verhalten	Relevante Methoden/Ansätze (Auswahl)
*Assessment*	Problemanalyse und Datenerhebung	Identifikation von Barrieren für klimagesundes Verhalten	Online-Umfragen, Experience Sampling, Fokusgruppen, Einzelinterviews
*Development*	Entwicklung von Maßnahmen	Gestaltung gezielter Klimaschutzinterventionen	Expert*innen-Workshops, Feldstudien, (Online‑)Experimente
*Implementation*	Umsetzung der Maßnahmen	Einführung von neuen Verhaltensförderungen	Nudging, Boosting, Policy-Interventionen
*Evaluation*	Überprüfung der Effektivität	Anpassung der Maßnahmen basierend auf Evaluation	(Online‑)Experimente, Langzeitstudien

Der Zyklus bindet Erkenntnisse der Verhaltensforschung systematisch ein. Dabei kann das Wissen aus dem COM-B-Framework eingesetzt werden, um im Rahmen des Action Cycle geeignete Maßnahmen zu planen und umzusetzen.

Obgleich es viele weitere Theorien und Modelle aus der Verhaltens- und Public-Health-Forschung gibt, veranschaulichen diese beiden Ansätze exemplarisch, an welchen Stellen verhaltenswissenschaftliche Erkenntnisse eingebunden werden können, um Interventionen effektiv und wissenschaftlich fundiert zu gestalten.

### Methodische Ansätze zur Datenerhebung

Die Untersuchung klimagesunden Verhaltens erfordert eine multi- und interdisziplinäre methodische Herangehensweise, indem (quantitative und qualitative) Methoden miteinander verknüpft werden (Mixed-Methods-Ansatz). Dies ist sinnvoll, da sich klimabezogenes Verhalten durch ein komplexes Zusammenspiel individueller, sozialer und struktureller Faktoren auszeichnet. Ein einzelnes methodisches Vorgehen kann diesen Facetten häufig nicht vollumfänglich gerecht werden. Durch die Kombination unterschiedlicher Verfahren lässt sich das Zusammenspiel dieser Faktoren umfassender abbilden und besser verstehen [17, 18].

Während quantitative Methoden dabei helfen, generalisierbare Muster zu erkennen, liefern qualitative Ansätze beispielsweise tiefere Einblicke in Entscheidungsprozesse und deren Kontext [19]. Die Kombination beider Perspektiven ermöglicht eine umfassendere Analyse und verbessert die Ableitung effektiver Public-Health-Interventionen [20, 21].

Verhaltensdaten können über verschiedene Verfahren erfasst werden. Nachfolgend werden überblicksartig einige ausgewählte Methoden eingeführt (vgl. [22] für Übersicht).

### Quantitative Methoden

1. *(Online‑)Umfragen* [23]: Diese Methode eignet sich zur großflächigen Erhebung von Einstellungen, Wissen und Selbstberichten über klimabezogenes Verhalten. Sie bietet den Vorteil einer hohen Reichweite und ermöglicht Vergleiche zwischen unterschiedlichen Populationen. Allerdings besteht das Risiko von Verzerrungen durch soziale Erwünschtheit und retrospektive Fehleinschätzungen sowie dadurch, dass nicht alle sozialen Gruppen online erreicht werden können.
2. *Experience Sampling *[24]: Durch diese Methode werden Verhaltensdaten in Echtzeit erfasst, indem Teilnehmende wiederholt kurze Fragen zu ihrem aktuellen Verhalten, ihren Emotionen und ihrer Umgebung beantworten. Dies reduziert Verzerrungen durch Erinnerungseffekte und ermöglicht eine präzisere Analyse kontextabhängiger Entscheidungsprozesse.
3. *(Online‑)Experimente* [22, S. 54–59]: Experimentelle Designs ermöglichen die gezielte Manipulation von Variablen, um kausale Zusammenhänge zwischen Interventionen und klimabezogenem Verhalten zu untersuchen. Online-Experimente sind besonders nützlich, da sie eine schnelle Rekrutierung von Teilnehmenden ermöglichen und unterschiedliche experimentelle Bedingungen kontrolliert getestet werden können. Auch hier ist jedoch zu beachten, dass sie gewisse Bevölkerungsgruppen ausschließen.
4. *Kooperationen mit Institutionen und externe Datenquellen:* Der Zugang zu bestehenden Datensätzen kann wertvolle Erkenntnisse liefern, insbesondere durch die Kombination von Umweltdaten (z. B. Satellitendaten zu Temperaturveränderungen) mit Befragungsdaten. Derartige interdisziplinäre Kooperationen ermöglichen eine differenziertere Betrachtung der Zusammenhänge zwischen (klimatischen) Umweltbedingungen und individuellen Verhaltensweisen. Externe Datensätze stammen z. B. aus Kooperationen mit dem Umweltbundesamt (UBA), dem Robert Koch-Institut (RKI) oder dem 2025 gegründeten Bundesinstitut für Öffentliche Gesundheit (BIÖG).

### Qualitative Methoden

- *Interviews* [25]: Durch halbstrukturierte oder offene Interviews können tiefergehende Einblicke in individuelle Entscheidungsprozesse, Wahrnehmungen und Motivationen im Zusammenhang mit klimagesundem Verhalten gewonnen werden. Diese Methode ermöglicht es, individuelle Perspektiven detailliert zu erfassen, ist jedoch zeitaufwendig und stark von der Gesprächsführung abhängig.
- *Fokusgruppen* [25]: Gruppeninterviews erlauben die Untersuchung sozialer Normen, kollektiver Meinungen und gemeinschaftlicher Barrieren im Bereich klimagesunden Verhaltens. Dabei können gruppendynamische Prozesse genutzt werden, um Diskussionen über Herausforderungen und Potenziale anzuregen.
- *Ethnografische Studien* [26]: Beobachtungen in natürlichen Kontexten helfen dabei, implizite Verhaltensmuster, soziale Interaktionen und kulturelle Einflüsse auf klimagesundes Verhalten zu identifizieren. Diese Methode ist besonders wertvoll für tiefgehende Analysen, erfordert jedoch einen hohen Zeit- und Ressourcenaufwand.

Abschließend sei angemerkt, dass die Grenzen zwischen quantitativen und qualitativen Methoden in der Praxis nicht immer strikt verlaufen. So kann etwa eine Tagebuchstudie als Experience-Sampling-Studie mit einer großen Teilnehmendenzahl und strukturiertem Fragebogenformat eher quantitative Auswertungsstrategien erfordern, während eine kleine, explorative Tagebuchstudie sich stärker an qualitativen Analysen orientiert. Entscheidend ist, dass alle Methoden in ihrer Konzeption und Durchführung den jeweiligen Forschungsfragen und Zielen angepasst werden.

Die zuvor dargestellten Methoden kommen in den Projekten HEATCOM und PACE (siehe Abschnitt „Fokus: Nutzung von Verhaltensdaten zur Förderung klimagesunden Verhaltens“) zum Einsatz, wo insbesondere repräsentative Online-Befragungen, Online-Experimente und Experience Sampling kombiniert werden, um klimagesundes Verhalten zu erfassen. Damit wird deutlich, wie ein projektspezifischer Methodenmix in der Public-Health-Forschung praxisnah umgesetzt wird.

## Fokus: Nutzung von Verhaltensdaten zur Förderung klimagesunden Verhaltens

Die systematische Erhebung und Auswertung von Verhaltensdaten ermöglichen es, auf die Herausforderungen und Chancen im Bereich klimagesunden Verhaltens zu reagieren. Auf Basis dieser Daten lassen sich sowohl individuelle als auch gesellschaftliche Prozesse differenzierter analysieren, um die Wirksamkeit von Public-Health-Strategien zu steigern und die bislang vorwiegend naturwissenschaftlich geprägten Daten (z. B. aus Epidemiologie, klinischer Medizin oder Umweltforschung) durch verhaltenswissenschaftliche Erkenntnisse zu ergänzen [27]. Im Folgenden werden zentrale Aspekte bei der Nutzung von Verhaltensdaten herausgearbeitet, wobei auf die Identifikation von Verhaltensmustern, die Analyse von Barrieren und Potenzialen sowie ein Praxisbeispiel zur Anwendung eingegangen wird.

### Identifikation von Verhaltensmustern

Verhaltensdaten liefern Einblicke in den Alltag der Menschen und decken auf, welche Routinen oder Gewohnheiten klimabezogenes Verhalten begünstigen oder hemmen [4]. Hierbei kann beispielsweise mithilfe von Online-Umfragen oder Experience Sampling untersucht werden, zu welchen Tageszeiten und unter welchen Umständen Menschen besonders empfänglich für klimafreundliche Handlungsoptionen sind. Durch eine Kombination verschiedener Methoden – etwa der Verknüpfung von Befragungsdaten mit Umweltdaten – entsteht ein detailliertes Bild davon, wie sich individuelle Entscheidungen in unterschiedlichen Kontexten (z. B. Wohnort, Arbeitssituation) gestalten [17].

### Praxisbeispiel: PACE und HEATCOM

Ein praxisnahes Beispiel für die Nutzung von Verhaltensdaten zur Förderung klimagesunden Verhaltens liefern die Projekte PACE (Planetary Health Action Survey) und HEATCOM (Verhaltensdaten für eine wirksame Hitzekommunikation), welche sowohl von den Universitäten Erfurt und Bamberg sowie dem Bernhard-Nocht-Institut für Tropenmedizin in Hamburg durchgeführt werden [5, 31–33].

PACE widmet sich dem klimagesunden Verhalten im Allgemeinen und erfasst relevante psychologische, soziale und strukturelle Einflussfaktoren. Das entwickelte PACE-Modell bietet somit eine Grundlage zur Analyse klimagesunden Verhaltens und dient als theoretisches Fundament, aus dem konkrete Konstrukte und Messinstrumente abgeleitet werden können.

**Abb. 1:**
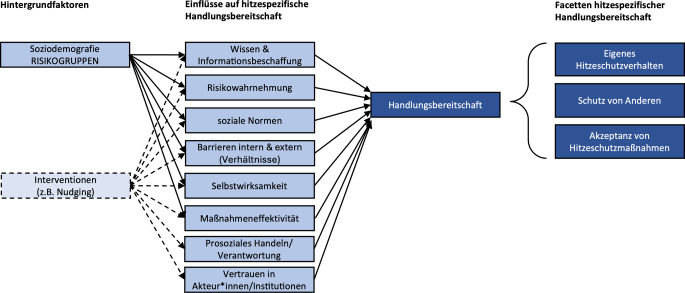
Im Projekt HEATCOM entwickeltes adaptiertes PACE-Modell zur Erfassung des Hitzeschutzverhaltens in der Bevölkerung. Eigene Abbildung inhaltlich angelehnt an [5]

Aufbauend auf den Erkenntnissen aus PACE fokussiert das Projekt HEATCOM das Hitzeschutzverhalten der Bevölkerung in Deutschland. In diesem Projekt wurde das PACE-Modell auf Hitzeschutzverhalten adaptiert (Abb. 1). Hier kommen sowohl quantitative als auch qualitative Ansätze zum Einsatz, um Faktoren zu identifizieren, die den Umgang mit hohen Temperaturen beeinflussen. HEATCOM analysiert unter anderem, wie stark sich Menschen durch Hitze beeinträchtigt fühlen, welche Hitzeschutzstrategien genutzt werden, warum Schutzmaßnahmen ggf. unterbleiben und welche Informationsquellen relevant sind.

Beide Projekte liefern wichtige Erkenntnisse über das Zusammenspiel von individuellen und strukturellen Einflussfaktoren und illustrieren, wie klimagesundes Verhalten in unterschiedlichen Kontexten gefördert werden kann.

### Identifikation und Adressierung von Barrieren

Verhaltensdaten ermöglichen die Identifikation von Barrieren, die Menschen daran hindern, klimagesunde Entscheidungen zu treffen. Diese Barrieren können *psychologischer Natur* sein [28], etwa mangelndes Wissen über gesundheitliche Risiken oder fehlendes Bewusstsein für den eigenen Einfluss auf das Klima.

Sie können aber auch *soziale* Dimensionen aufweisen [29], beispielsweise wenn traditionelle Normen oder Gruppendruck den Wandel zu umweltbewussten Verhaltensweisen erschweren.

Hinzukommen *strukturelle Faktoren* (Bedingungen der Umwelt; [28, 30]), wie unzureichend ausgebaute Fahrradwege, fehlende schattige Orte während Hitzeperioden oder mangelnde Verfügbarkeit gesunder und nachhaltiger Lebensmittel.

An dieser Stelle ist es wichtig, erneut das Zusammenspiel von Verhaltenswissenschaft und verhältnisorientierten Ansätzen zu betonen [9, 11]: Nur wenn individuelle Verhaltensänderungen durch geeignete Rahmenbedingungen flankiert werden, lassen sich nachhaltige Effekte erzielen [4]. Ein einseitiger Fokus auf individuelles Verhalten vernachlässigt die notwendigen strukturellen Anpassungen, während rein strukturelle Maßnahmen ohne Berücksichtigung individueller Motivationen und Fähigkeiten ebenfalls ihre Wirkung verfehlen können.

Auf Basis der aus HEATCOM und PACE gewonnenen Erkenntnisse wird deutlich, dass Verhaltensdaten aufzeigen können, welche psychologischen, sozialen oder strukturellen Hürden Menschen daran hindern, klimagesunde bzw. hitzeschutzbezogene Entscheidungen zu treffen. Typische Beispiele dafür sind:

- *mangelndes Wissen* über gesundheitliche Risiken und klimabezogene Zusammenhänge,
- *soziale Normen*, die umweltfreundliches Verhalten in bestimmten Gruppen nicht unterstützen,
- *infrastrukturelle Defizite*, etwa fehlende Beschattung im öffentlichen Raum oder unzureichende ÖPNV-Anbindung.

Durch gezielte Interventionen, wie Informationskampagnen, Nudging- (Interventionen zur Unterstützung von Entscheidungen durch Gestaltung der Entscheidungsumgebung) oder Boosting-Ansätze (d. h. Maßnahmen zur Stärkung individueller Kompetenzen und Entscheidungsfähigkeiten) sowie politische Maßnahmen zur Verbesserung der umweltfreundlichen Infrastruktur, lassen sich diese Barrieren schrittweise abbauen [4, 34, 35].

## Potenziale und Herausforderungen

Die bisherigen Ausführungen zeigen, dass Verhaltensdaten einen wichtigen Beitrag zur Förderung klimagesunden Verhaltens leisten können. Ihre Nutzung ist mit vielfältigen Potenzialen, allerdings auch mit erheblichen Herausforderungen verbunden. Dieser Abschnitt zeigt auf, wie Verhaltensdaten evidenzbasierte Public-Health-Strategien stärken und welche ethischen, datenschutzrechtlichen und interdisziplinären Fragen sich dabei stellen.

### Chancen durch gezielte Datennutzung

Der Einsatz von Verhaltensdaten ermöglicht eine *verbesserte Zielgruppensegmentierung* und *individualisierte Ansprache* [36, 37]. Statt „One-size-fits-all“-Maßnahmen einzusetzen, können Interventionen (inklusive der kommunizierten Botschaften) passgenau auf bestimmte Bevölkerungsgruppen zugeschnitten werden, was ihre Effektivität erheblich steigern kann.

Darüber hinaus bietet die *kombinierte Auswertung *von Daten aus verschiedenen Quellen – etwa Sozialwissenschaften, Umweltforschung und Epidemiologie – die Chance, komplexe Wechselwirkungen zwischen individuellen und strukturellen Faktoren besser zu verstehen [38].

Nicht zuletzt kann die kontinuierliche Erhebung von Verhaltensdaten eine *dynamische Anpassung *von Interventionen ermöglichen: Durch regelmäßige Evaluationen werden Maßnahmen frühzeitig optimiert und an veränderte Rahmenbedingungen angepasst [4, 39].

### Ethische Erwägungen und Datenschutz

Die Verarbeitung sensibler Verhaltensdaten wirft notwendigerweise ethische Fragen auf. Insbesondere Aspekte des Datenschutzes, der Datensicherheit und der Einwilligung zur Datenerhebung müssen bedacht werden. Ohne eine solide Rechtsgrundlage und transparente Kommunikation kann das Vertrauen der Bevölkerung in Public-Health-Maßnahmen nachhaltig geschädigt werden [40–42]. Eine verantwortungsvolle Datennutzung setzt voraus, dass Erhebungs- und Auswertungsverfahren klar definiert und den Teilnehmenden transparent offengelegt werden. Zudem sollte geprüft werden, inwieweit Anonymisierungs- und Pseudonymisierungsverfahren eingesetzt werden können, um den Schutz der Privatsphäre zu gewährleisten.

### Interdisziplinäre Perspektiven

Um das volle Potenzial von Verhaltensdaten auszuschöpfen, bedarf es einer engen Kooperation zwischen Public Health, Umweltwissenschaften und Verhaltensforschung [27, 43]. Die Betrachtung klimabezogenen Verhaltens erfordert ein breit angelegtes Fachwissen, das naturwissenschaftliche, gesellschaftliche und psychologische Aspekte miteinander verbindet. Nur durch eine systematische Verzahnung dieser Disziplinen können wirkungsvolle Maßnahmen effektiv entwickelt werden.

### Manipulationsvorwurf und wissenschaftliche Evidenz

Es besteht häufig die Sorge, verhaltenswissenschaftliche Maßnahmen könnten Menschen unzulässig beeinflussen oder gar bevormunden. Hier ist zu betonen, dass es in der Public Health primär um den Erhalt bzw. die Förderung der Gesundheit geht und nicht darum, Verhaltensweisen ohne entsprechende medizinische oder wissenschaftliche Evidenz durchzusetzen [4]. Vielmehr wird angestrebt, die Entscheidungsfreiheit der Individuen zu wahren und evidenzbasierte Informationen transparent zu kommunizieren. Interventionsformen wie Nudging oder Boosting zielen darauf ab, gesundheitsförderliche Entscheidungen zu erleichtern, ohne gesetzlichen Zwang auszuüben [34, 44].

Entscheidend ist die Transparenz darüber, in welcher Form Verhaltensdaten erhoben und ausgewertet werden und wie sie in Maßnahmen einfließen. Eine solche Herangehensweise stellt sicher, dass Manipulation nicht das Ziel ist, sondern vielmehr ein verantwortungsvoller, partizipativer und wissenschaftlich fundierter Zugang zu Gesundheits- und Klimaschutzthemen.

Der Vorwurf der Manipulation ist besonders beim Boosting fehlgeleitet: Dieser Ansatz ist dem Betroffenen gegenüber komplett transparent, da z. B. Fähigkeiten gestärkt werden.

### Herausforderungen in der politischen Umsetzung

Obwohl Verhaltensdaten ein großes Potenzial bergen, ist ihre Integration in politische Entscheidungsprozesse keineswegs trivial [4, 9, 45]. Zum einen sind verwaltungstechnische Hürden zu bewältigen, wenn es darum geht, Daten aus unterschiedlichen Quellen zusammenzuführen. Zum anderen besteht das Risiko, dass die Bedeutung von Kontextfaktoren unterschätzt wird, wenn politische Maßnahmen zu stark auf individuelle Verhaltensänderungen abzielen. Daher ist es wesentlich, systemische Veränderungen vom Menschen her zu planen, indem Verhaltens- und Verhältnisänderungen gleichwertig berücksichtigt werden.

Zudem müssen politische Entscheidungsträger*innen angemessen über die Potenziale und Grenzen der Verhaltensdaten informiert sein, um sinnvolle Maßnahmen zu ergreifen [13]. Das Spektrum reicht dabei von klaren Kommunikationsstrategien, die beispielsweise auf Nudging oder Boosting setzen, bis hin zu institutionellen Reformen, die den strukturellen Wandel fördern.

Mit einer transparenten, partizipativen Vorgehensweise kann auch dem Vorwurf der Manipulation begegnet werden, da klar ersichtlich wird, in welchem Rahmen und zu welchem Zweck Daten erhoben und genutzt werden.

## Fazit und Handlungsempfehlungen

Die Ausführungen der vorangegangenen Abschnitte verdeutlichen, wie wertvoll die systematische Nutzung von Verhaltensdaten zur Förderung klimagesunden Verhaltens sein kann. Gleichzeitig wird klar, dass neben individuellen Faktoren auch strukturelle Bedingungen entscheidend sind, damit nachhaltige Verhaltensweisen langfristig umgesetzt werden können.

Entscheidend ist dabei die konsequente Verbindung von Verhaltens- und Verhältnisansätzen: Individuelle Motivation und Wissen allein genügen nicht, wenn entsprechende Infrastrukturen und politische Rahmenbedingungen fehlen. Umgekehrt laufen strukturelle Reformen ins Leere, wenn sie nicht die tatsächlichen Bedarfe und Motivationen der Bevölkerung berücksichtigen.

### Integration in Public-Health-Strategien

Für politische Entscheidungsträger*innen bietet sich die Chance, Verhaltensdaten als entscheidendes Fundament für neue Strategien zu nutzen. Eine klare Priorisierung besteht dabei darin, Informations- und Sensibilisierungskampagnen mit strukturellen Maßnahmen zu verknüpfen. Beispielsweise lassen sich klimagesunde Mobilitätsalternativen (z. B. ÖPNV-Ausbau) effektiver in der Bevölkerung verankern, wenn gleichzeitig durch Kommunikationsinitiativen die Vorteile dieser Alternativen hervorgehoben werden.

Die partizipative Einbindung relevanter Stakeholder – wie Umweltverbände, Gesundheitsinstitutionen oder Bürger*innen-Initiativen – kann sicherstellen, dass Interventionen bedarfsgerecht und akzeptanzfördernd gestaltet werden [46, 47]. Zudem können entsprechende Taskforces eingerichtet werden und beratend tätig sein. Eine weitere Initiative, die an dieser Stelle exemplarisch erwähnt werden kann, ist das Behavioural-Science-Connect-Netzwerk, in welchem sich Forscher*innen aus den Sozial- und Verhaltenswissenschaften organisieren und gemeinsame Stellungnahmen und Beratungen unter anderem für politische Entscheider*innen koordinieren [48].

Insgesamt sollten, ganz im Sinne einer evidenzbasierten Gesundheitskommunikation, (kommunikative) Public-Health-Strategien immer wieder anhand neuer Erkenntnisse und aktueller Verhaltensdaten evaluiert und angepasst werden, um auf Veränderungen in der Gesellschaft und der Umwelt dynamisch reagieren zu können.

### Forschungsperspektiven

Trotz bestehender Studien, wie HEATCOM und PACE, gibt es noch zahlreiche offene Fragestellungen und Themen, die zukünftig weiterverfolgt werden sollten. Dazu zählen:

- *Langfristige Wirkung:* Welche Faktoren sind entscheidend, damit klimagesundes Verhalten auch über kürzere Aktionszeiträume hinaus beibehalten wird?
- Nutzer*innenzentrierte Datenintegration: Wie können Verhaltensdaten aus unterschiedlichen Quellen so zusammengeführt werden, dass sie aussagekräftige, praxisnahe Handlungsempfehlungen liefern?
- *Technologische Entwicklungen:* Welche Rolle spielen digitale Tools, etwa Apps oder Wearables, bei der Erfassung und Förderung klimagesunden Verhaltens?
- *Globale und kulturelle Kontexte:* Wie lassen sich Ansätze aus Deutschland oder Europa auf andere Länder mit unterschiedlichen kulturellen und infrastrukturellen Bedingungen übertragen?

Eine stärkere Vernetzung zwischen Akteur*innen aus Wissenschaft, Politik und Praxis kann maßgeblich dazu beitragen, diese Forschungsfragen zu adressieren und evidenzbasierte Maßnahmen zu entwickeln.

### Konkrete Handlungsempfehlung

Abschließend lässt sich festhalten, dass klimagesundes Verhalten erst dann breitenwirksam etabliert werden kann, wenn alle beteiligten Akteur*innen – von Forschung und Politik über Institutionen bis hin zu den Bürger*innen selbst – an einem Strang ziehen. In diesem Prozess nehmen Verhaltensdaten eine Schlüsselstellung ein: Sie liefern die Basis, um Maßnahmen kontinuierlich zu optimieren und an gesellschaftliche Veränderungen anzupassen.

Zusammengefasst lautet die zentrale Handlungsempfehlung dieses Beitrags: Politische Entscheidungsträger*innen und Public-Health-Institutionen sollten die systematische Erhebung und Nutzung von Verhaltensdaten als festen Bestandteil ihrer Strategien zur Förderung klimagesunden Verhaltens etablieren. Dabei gilt es, interdisziplinäre Kooperationen zu stärken, den Aufbau entsprechender Forschungs- und Anwendungsstrukturen nachhaltig zu fördern und Kommunikationsmaßnahmen evidenzbasiert an Bedarfen auszurichten.
